# Adhesive assisted TiB_2_ coating effects on friction stir welded joints

**DOI:** 10.1038/s41598-022-21281-6

**Published:** 2022-10-25

**Authors:** Amit Kumar Kundu, Manoj Kumar Gupta, Nitesh Singh Rajput, Rajesh Rathore

**Affiliations:** 1 Mechanical Engineering Department, Amity University Rajasthan, Jaipur, India; 2 Mechanical Engineering Department, Hemvati Nandan Bahuguna Garhwal University, Srinagar, Uttarakhand India

**Keywords:** Engineering, Materials science

## Abstract

Friction stir welding is a novel technique for joining ferrous and non-ferrous materials in a solid state. The groove fill techniques are most popular and generally used by researchers to dope reinforcement in the FSWed zone to improve the properties of joints. The main drawback of this technique is that a few amounts of reinforcement material come out from the groove during the fabrication of the joint. In the present work, the adhesive-assisted reinforcement technique was used to overcome this problem for the fabrication of particulates reinforced friction stirred weld joint. In the present work, the aluminum alloy plate edges were coated with a thin layer of TiB2. The coated and non-coated edge plates were joined using friction stir welding at the rotational speed of 1400 and 2240 rpm, and welding speed of 32 mm/min using a taper threaded pin tool. The tensile strength of coated edge plate welded joints was found highest in comparison to non-coated joints which was 39.74% superior. The percentage elongation of coated edge joint was observed about 1.5 times lower than the non-coated edge plate joint. The flexure strength of TiB2 reinforced coated edge joint was found about 1.5 times higher. However, the impact strength of coated edge plate was found nearly three times lower than the uncoated edge joints. The TiB2 coated edge joints reveal 22.75% higher hardness than the non-coated edge plate joints welded at the rotational speed of 2240.

## Introduction

Friction stir welding (FSW) is a solid-state joining process that uses a non-consumable tool to join two facing workpieces without melting the workpiece material^1–3^. The heat is generated by friction between the rotating tool and the workpiece material, which leads to a softened region near the FSW tool. While the tool is traversed along the joint line, it mechanically intermixes the two pieces of metal, and forges the hot and softened metal by the mechanical pressure, which is applied by the tool. It has been reported that 87% of the heat is generated by the FSW tool shoulder because of friction between the shoulder surface and the workpiece^4^. The tool shoulder geometries affect the material flow during welding. The majority of tool shoulder has concave, convex, and flat profiles^5–12^. Hot-worked tool steel H13 is mostly used for welding aluminium alloys^10,13^. Tool tilt angle squeeze plastically deformed material beneath the tool. Generally, a tool tilt angle between 1° and 4° is used in FSW. FSW is capable of joining similar and dissimilar metals such as aluminium alloys, copper alloys, titanium alloys, mild steel, stainless steel, and magnesium alloys^14–18^. Even if FSW is a solid state welding process it generates significant heat input, leading to possible changes in the microstructure. The excessive aging or hardening within the nugget zone (NZ) area, the thermo-mechanically affected area (TMAZ), or the heat-affected zone (HAZ) of FSWed joints has been reported to occur^19,20^. Loss of mechanical properties is often seen in these changing areas of microstructure, especially in TMAZ and HAZ^21–24^. Mardalizadeh et al.^25^ reported that joints formed of AA2024 have lower hardness in HAZ and TMAZ. In spite of the optimization in welding parameters and cooling parameters during welding, the mechanical performance of FSWed joints remains lower than that of base metals because of the emergence of microstructure and mechanical properties due to the strong thermo-mechanical pressures found in the FSW process^26,27^.The FSWed joint properties mainly depend on the process parameters such as tool rotational speed, transverse speed, tool tilt angle, and plunge depth. The optimum values of the FSW parameters depend on the workpiece material properties, thickness, and tool geometry^28^. The hardness of the joint area increases as the tilt angle increases^29^. Elyasi et al.^30^ reported that the maximum tensile strength of joints was in the joining of aluminium alloy at 2° tilt angle in comparison to 1° and 3°. A similar observation was reported by Acharya et al. ^31^. The combination of tool rotational and transverse speed in FSW is complex because increasing the rotation speed or decreasing the traverse speed will result in a hotter weld and vice versa^32^. The microstructure and hardness of joints strongly depend on the rotational speed in comparison to the welding speed. Ghada et al.^33^ reported that the hardness of joints increased with the decrease in the rotational speed. Ganesh and Kumar^34^ investigated the superplasticity of friction stir welded aluminium alloy sheets at varying tool rotation speeds. The result shows that superplasticity significantly improved with the increase of tool rotation speed. It was observed that with the presence of reinforcement particles inside the nugget zone, the mechanical properties of the weld joint improved significantly. The reinforcing techniques used to reinforce reinforcement materials during friction stir welding in a friction stir weld zone are one of the key issues in FSW. The reinforcing techniques demine the volume and distribution of reinforcement materials in the friction stir weld zone during FSW. The properties of reinforced friction stirred weld also depend on the effective doping and distribution of reinforcement material in the friction stir weld zone. Saeidi et al.^35^ used the groove fill technique to fill Al_2_O_3_ nanoparticles for the fabrication of Al_2_O_3_ reinforced joints. They noticed that the corrosion resistance of the Al_2_O_3_ reinforced FSW joint was superior. However, the impact strength was lower due to weak bonding between parent and reinforcement materials. A similar technique was also used by Kumar et al.^36^ to introduce SiC and Si3N4 particulates in the friction stir weld zone. Dragatogiannis et al.^37^ machined a rectangular grove whose depth was half of the plate depth along the joint line for the fabrication of TiC reinforced friction stir weld joint. They reported that the hardness of TiC reinforced joints increased 18%. Furthermore, the tensile strength and ductility of joints also improved. The V-grooves technique was used by Huang^38^ to introduce iron-based reinforcement particulate in the friction-stir weld zone. The result reveals that the tensile strength and ductility of iron reinforced joint was inferior. However, the tensile and ductility increased with the increase of tool rotational speed. Singh et al.^39^ used hole fill techniques to incorporate Al_2_O_3_ particulates at the faying edges of stirred weld. They found that the hardness of joints increased with the increase of the volume fraction of Al2O3. Pantelis et al.^40^ reinforced SiC nano-particles in the FSW zone in welding of aluminium alloy. They reported that the hardness of the weld nugget was improved by 18% in comparison to without SiC addition. Pasha et al.^41^ investigated the mechanical behavior of varying percentages of SiC and Al_2_O_3_ reinforced welded joints of aluminium alloy. It was found that the tensile strength and hardness of SiC reinforced welded joints were superior in comparison to Al_2_O_3_ reinforced joints. However, ductility and impact strength of particulates reinforced joints showed inferior as compared to unreinforced welded joints.

Mostly groove fill techniques were used by the researcher to introduce reinforcement in FSWed zone. In grove fill techniques, there is a chance of reinforcement material comes out from the groove during the fabrication of the joint. In the present work, the adhesive-assisted reinforcement technique was used to minimize this kind of difficulty for the fabrication of particulates reinforced friction stirred weld joint. In this work, TiB_2_ powder was reinforced in a friction stir weld zone by coating the edged of the plate before welding which required to joint. The coating material was prepared by mixing Araldite adhesive and TiB_2_ powered in the ratio of 1:1. The Al 1120 alloy palate edges (6 mm X 120 mm) were coated with a thin layer of this coating material. The coated-edged plates were joined using a friction stir welding process at tool rotational varying tool rotational speed and constant welding speed 32 mm/min with the help of a taper threaded pin tool. The tensile strength, flexure strength, percentage elongation, impact strength, and hardness of joints were investigated and reported in this paper.

## Materials and methods

The commercial aluminium alloy Al 1120 flat of thickness 6 mm, width 55 mm, and length 100 mm was used for the preparation of the sample. The chemical composition of Al 1120 is shown in Table 1. The TiB_2_ powder and Araldite adhesive materials were for preparing coating material (Reinforcement material). The particle size of TiB_2_ powder was 325 mesh and purity and 99.9%.

**Table 1:** Chemical composition of Al 1120.

Material	Constituents (weight %)						
Al 1120	Si	Mg	Ca	Fe	Cu	Mn	Al
	0.18	0.26	0.12	0.43	0.22	0.03	98.76

**Figure 1:**
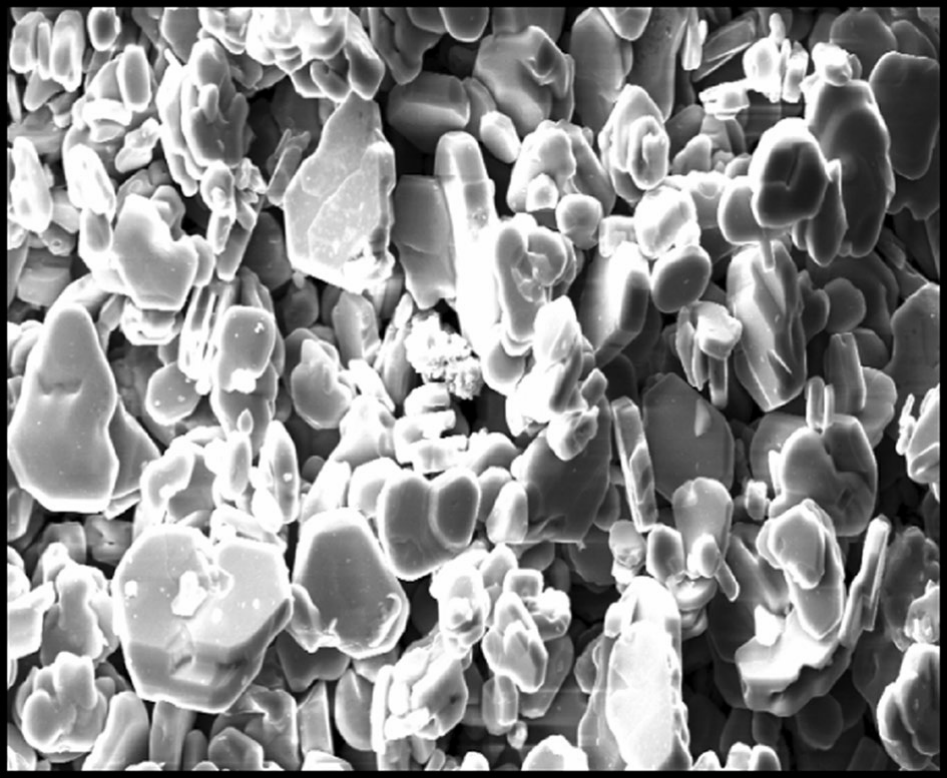
SEM image of TiB_2._

The SEM (Scanning electron microscopy) image of TiB_2_ is depicted in Fig. 1.

**Figure 2:**
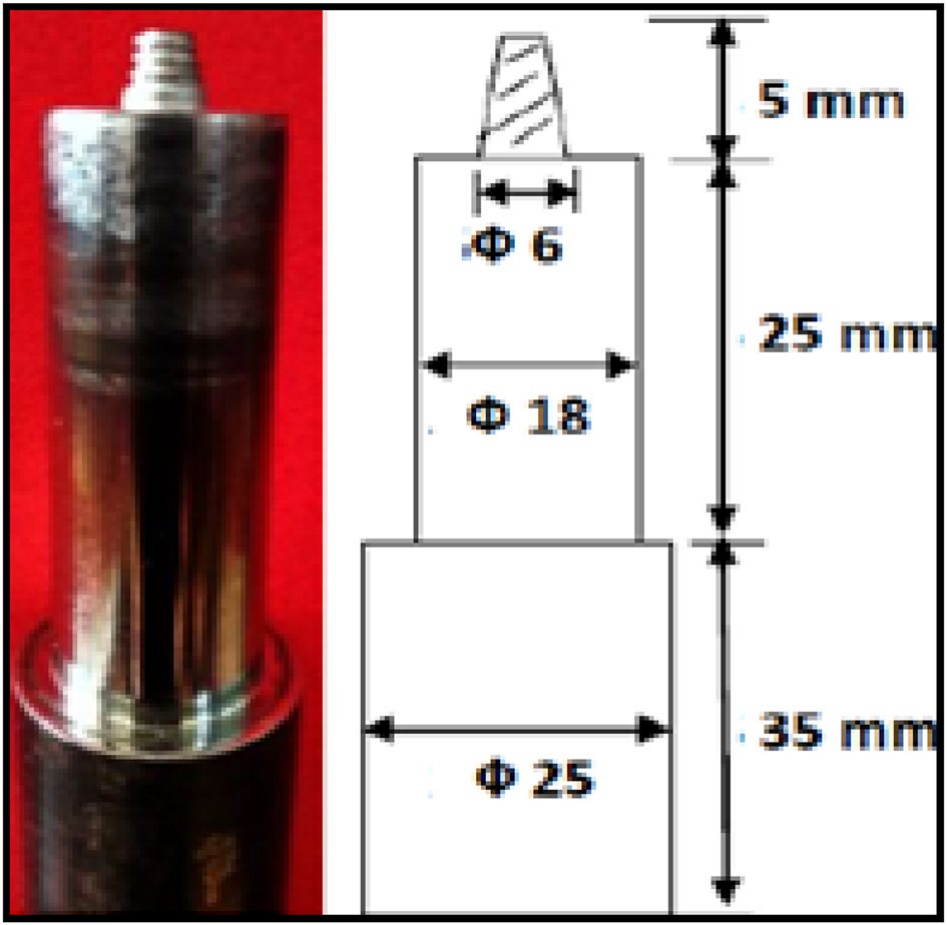
Taper threaded pin FSW tool.

**Figure 3:**
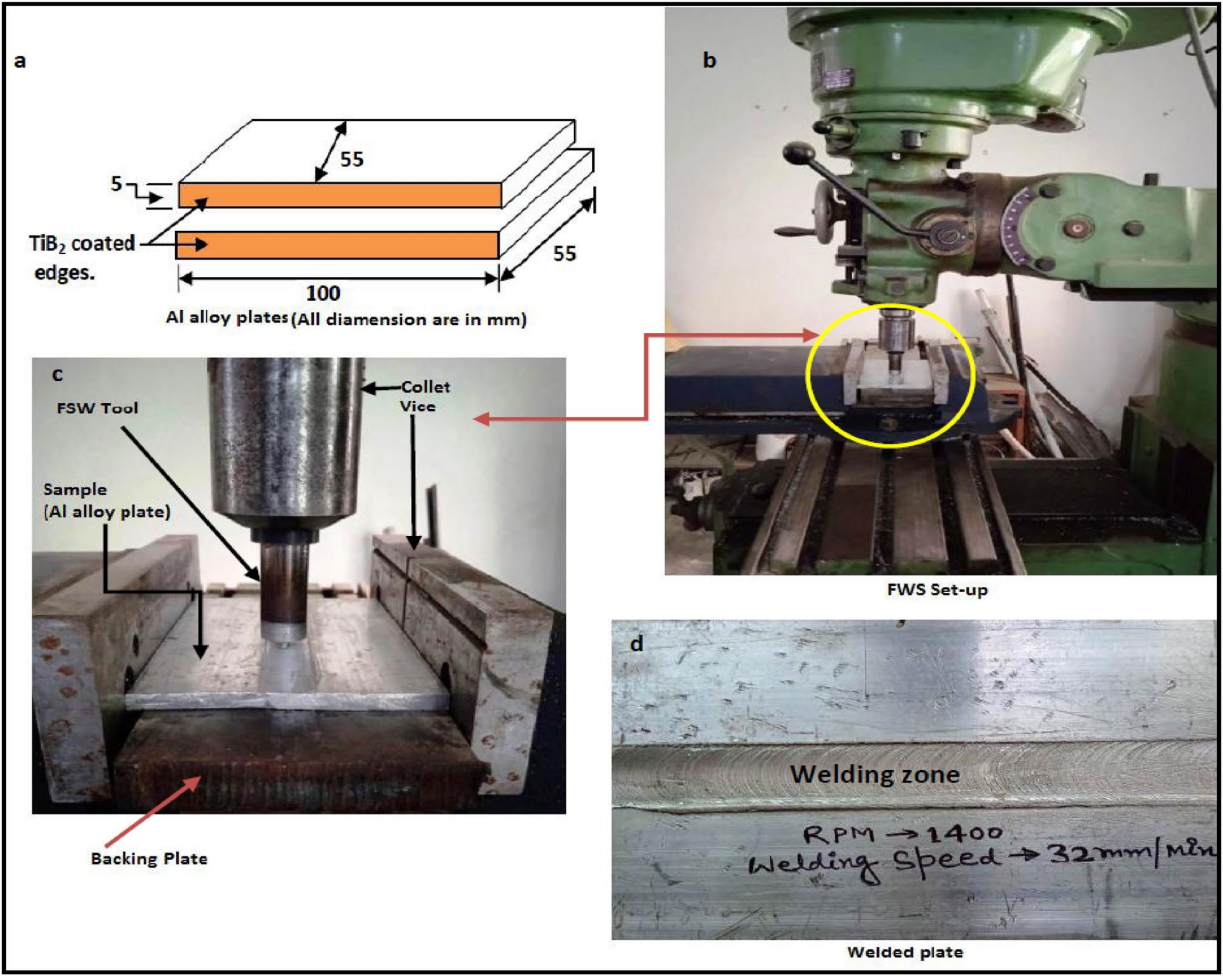
(**a**) Sample specification (**b**) FWS Set-up (**c**) Welding area (**d**) Welded plates.

The Friction Stir Welding setup was developed on a vertical milling machine (M1TR, HMT Limited, Pinjore, India) as shown in Fig. 3b. The cylindrical threaded profile tool made of H13 as shown in Fig. 2 was used for the fabrication of joints.

**Figure 4:**
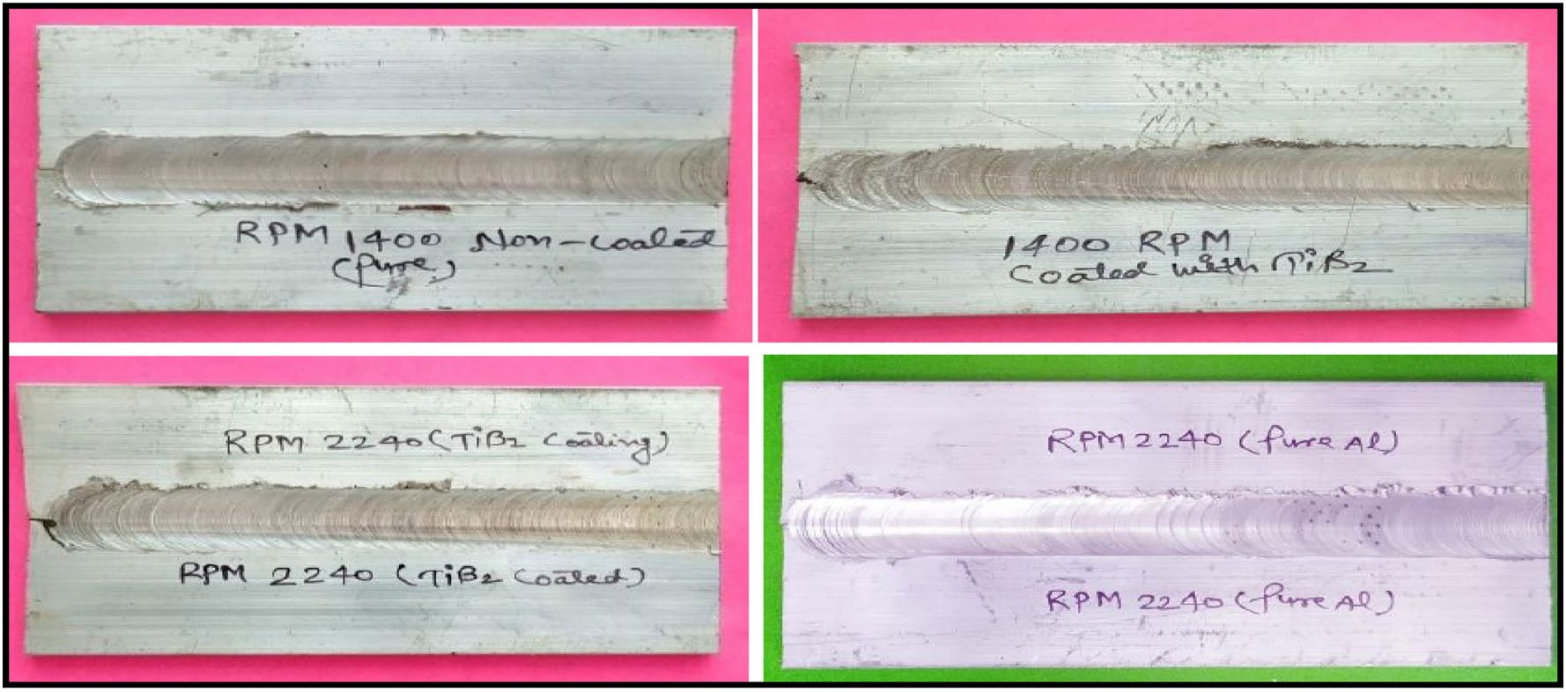
Fabricated joints of all samples.

**Figure 5:**
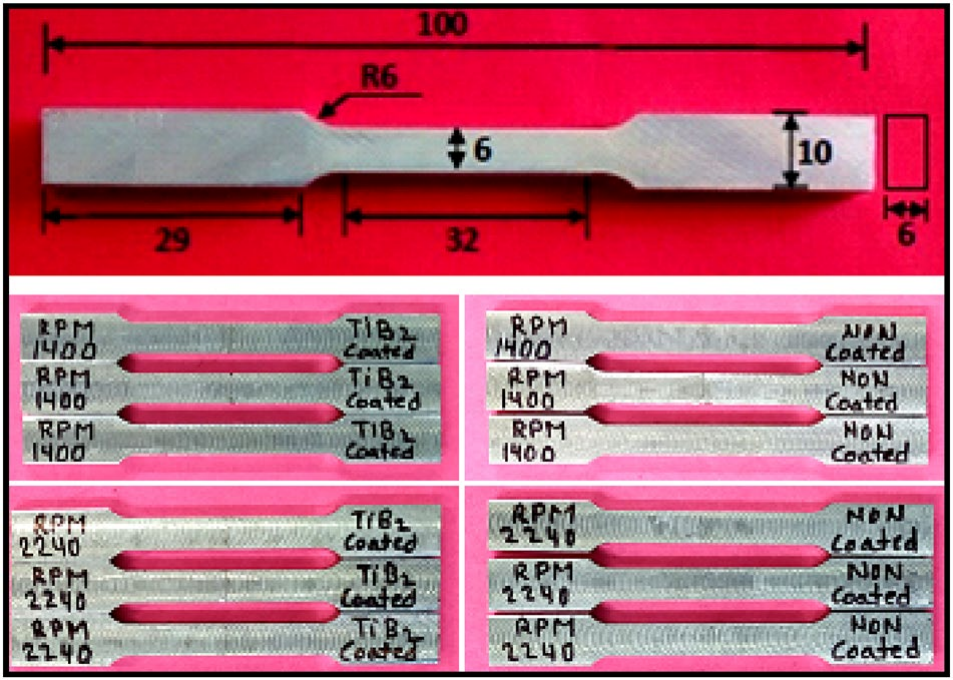
Tensile testing samples and dimension.

**Figure 6:**
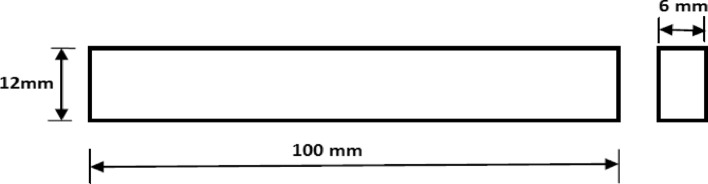
Dimension of flexure test sample.

**Figure 7:**
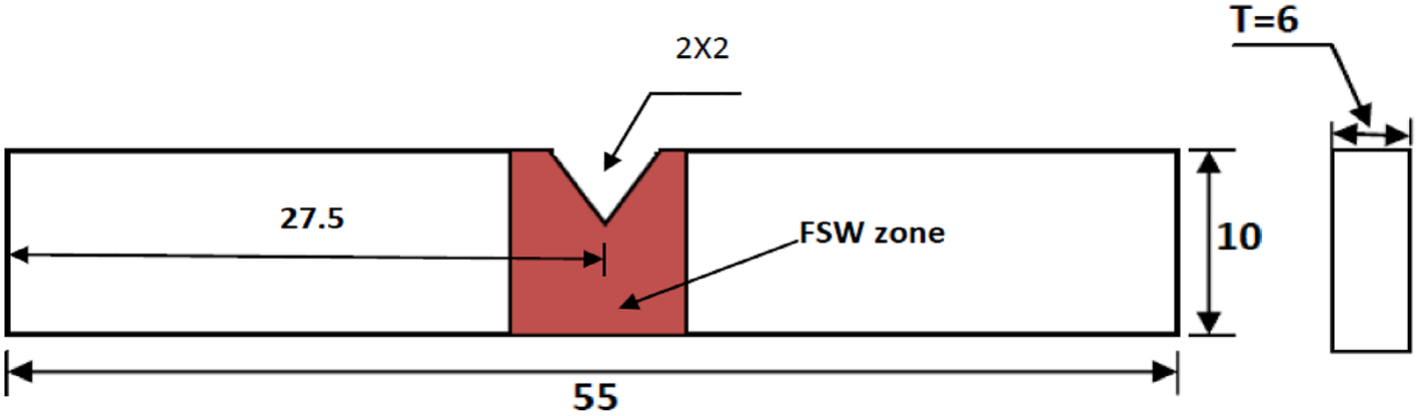
Dimension of impact test sample.

The welding parameters such as tool rotational speed 1400 rpm and 2240 rpm, and transverse speed 32 mm/min were selected for the fabrication of joints. The coating material was prepared by mixing Araldite adhesive and TiB2 powder in a ratio of 1:1. After that, a thin layer spread along the edges of each plate which is required to join and allows it to dry in an open atmosphere as shown in Fig. 3a. The thickness of the coating was approximately 0.3 mm on each plate edge. Further, the samples were firmly clamped in the butt position inside the milling vice on the backing plate as shown in Fig. 3c. Initially, the FSW parameters i.e. tool rotational speed was selected as 700, 900, 1400, 2240 rpm, and 32 mm/min travel speed. It was found that the joints fabricated at 700 and 900 rpm have voids (welding defects) in weld zone. The causes of this defect may be due to improper frictional heat generation at lower tool rotational speed. The FSW joints were fabricated at tool rotational speeds 1400 rpm and 2240 rpm, and welding speed 32 mm/min using the FSW tool as shown in Fig. 3. The welded sample is depicted in Fig. 3d. All categories of welded samples are depicted in Fig. 4. The testing samples for the tensile test were prepared as per the ASTM standard E8/E8M-09. The dimension and testing samples are shown in Fig. 5. The flexure testing sample was prepared as per the ASTM standard E290. The dimension is shown in Fig. 6. The computerized UTM machine (Neelam Engineering Company, Agra, India.) was used for performing tensile and flexure tests of joints. The tensile and flexure testing of welded samples was performed at the strain rate of 0.1 mm/min. The impact simple was prepared as per ASTM standard E-23 is shown in Fig. 7. The Charpy impact test was performed using a digital impact tester (Faune Test Equipment Pvt Ltd, Type- AIT-300D). Three samples of each category of welded samples were tested. The Vickers hardness tests of composites were conducted at a load of 5 kg and a dwell time 20 s. Three readings of hardness at different places in the FSWed zone were taken.

## Results and discussion

**Figure 8:**
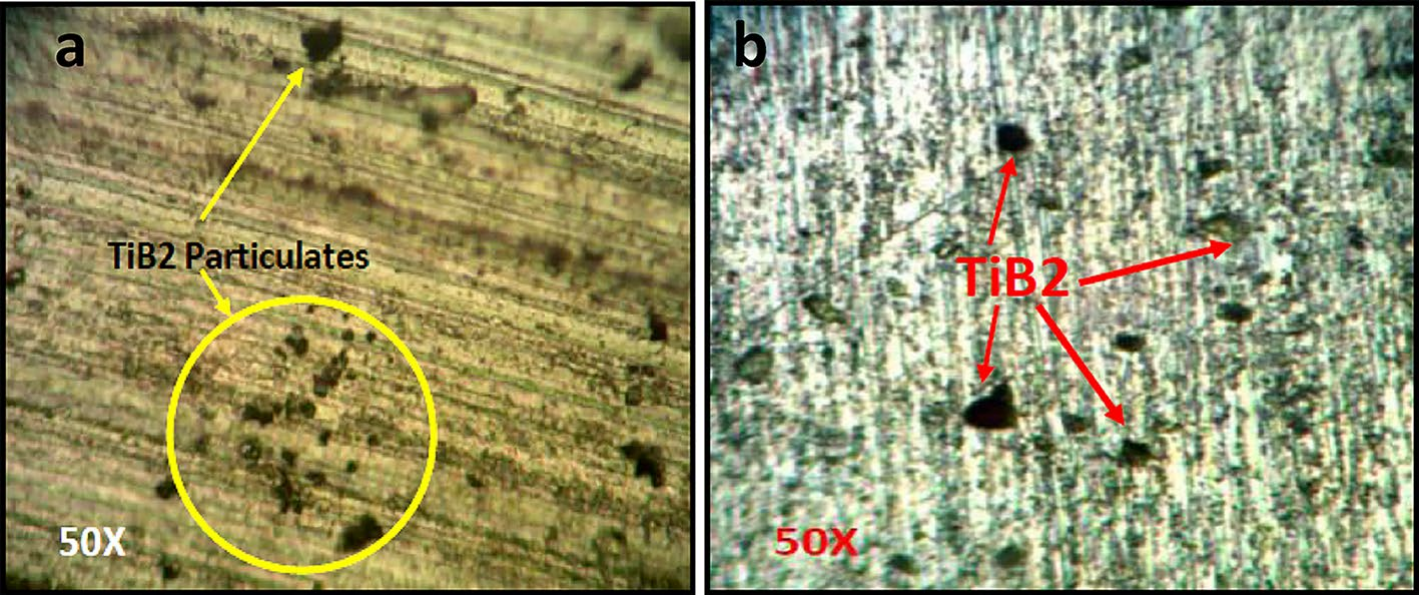
Optical micrograph of friction stir weld zone fabricated at (**a**) 2240 and (**b**) 1400 rpm.

**Figure 9:**
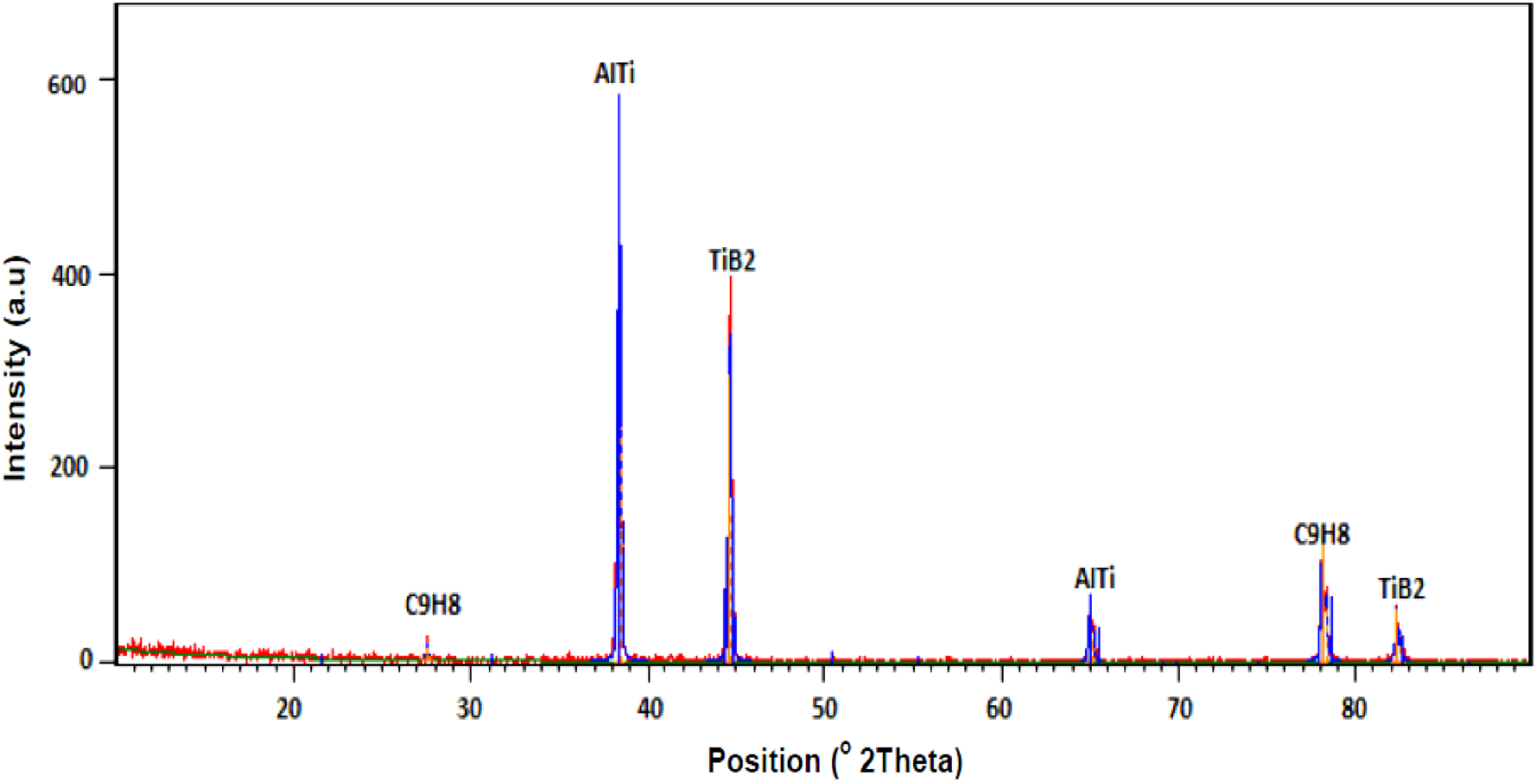
Energy dispersive X-ray analysis of TiB_2_ coated plate joint.

The successful welding joints of coated and non-coated edge plates were fabricated at 1400 and 2240 rpm and welding speed 32 mm/min. The optical microscopic was carried out to observe the presence and distribution of TB_2_ particulates in the friction stir welded zone. For the micrographic examination, the sample was prepared by cutting the material of welded zone, grinding, and polishing with different grades of emery paper using a double disk metallographic polishing machine. The micrograph of TiB2 coated edge joints of friction stir weld zone was taken at 50X and depicted in Fig. 8. Figure 8 shows the presence and distribution of TiB2 particulate in a friction stir weld zone fabricated at 2240 and 1400 rpm. The Energy Dispersive X-Ray Analysis (EDX) of the friction stir weld zone was carried out to find that the phase of Al, TiB2, and Araldite adhesive present in coated edge friction stir welded sample. The XRD test analysis report is depicted in Fig. 9. Figure 9 shows the peak of AlTi, TiB_2_, and C9H8 (Araldite) which conform to the presence of TiB_2_ in the friction stir weld zone.

### Tensile and flexure strength

**Figure 10:**
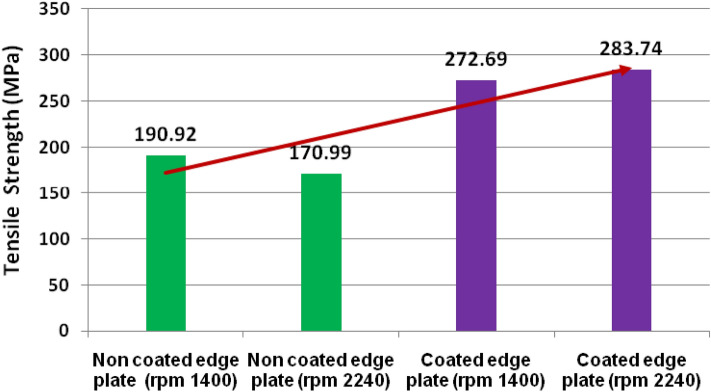
Tensile strength of FSW joints at varying rpm.

**Figure 11:**
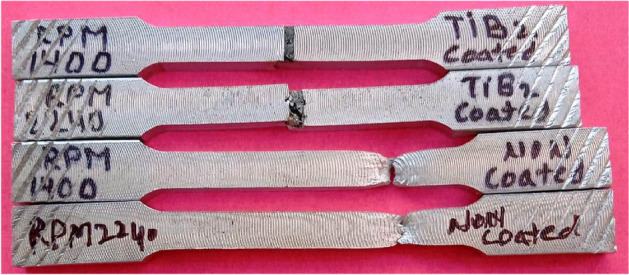
Tensile tested samples.

**Figure 12:**
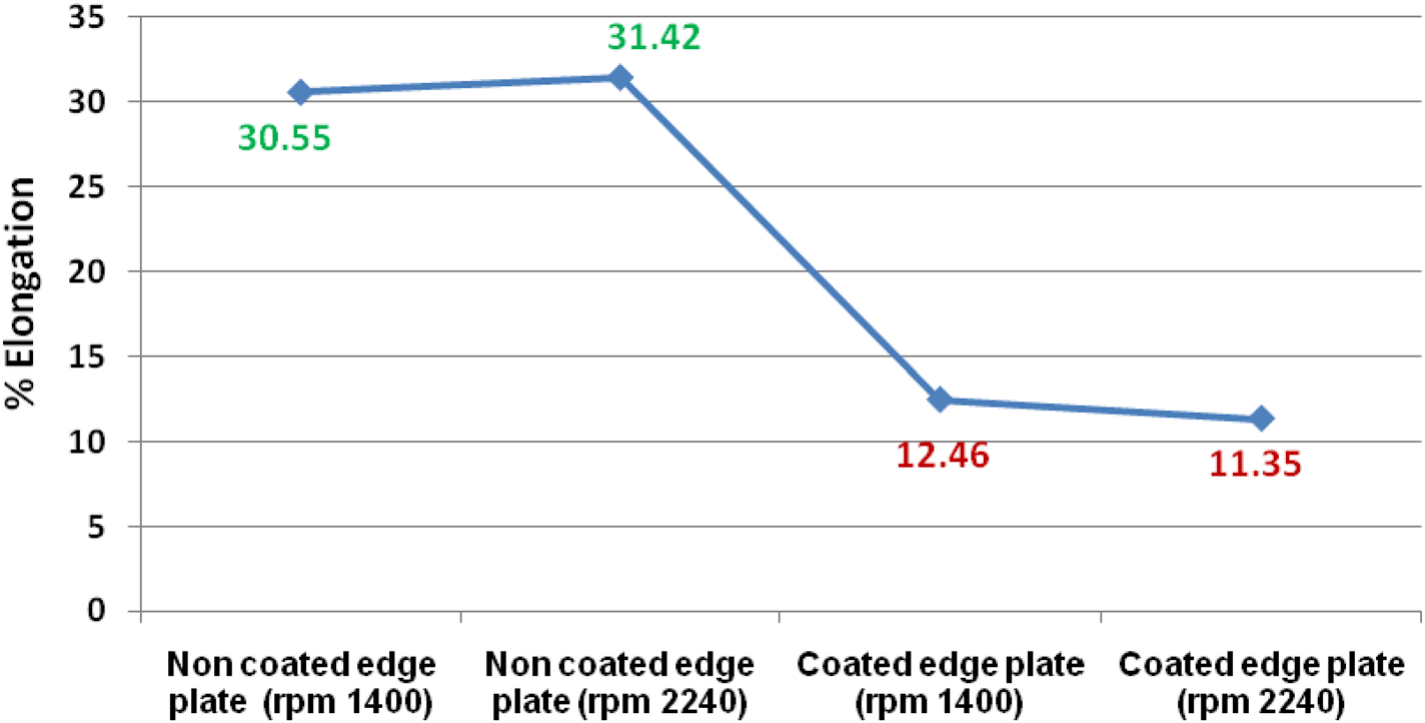
The % Elongation of welded joints at varying rpm.

The average value of three samples of each category of tensile and flexure strength was taken and reported in Table 2. The result of tensile strength and tested samples of each category are depicted in Figs. 10 and 11 respectively. The percentage elongation of tested samples is depicted in Fig. 12. Figure 10 reveals that the tensile strength of coated edge (TiB2 reinforced) joint is higher than the non-coated (unreinforced) edge joint due to the presence of TiB2 in the welded zone^36,37,39,41,42^. Furthermore, the tensile strength of the non-coated edge sample welded at the rpm of 1400 exhibits higher strength than the sample welded at 2240 rpm. However, coated plates welded at 2240 rpm showed superior strength as compared to those welded at 1400 rpm. The tensile strength of coated edge plate welded at 1400 rpm found that 30% higher than the non-coated edge plate joint welded at the same rpm. However, the coated plate joint at 2240 rpm showed 39.74% superior that the non-coated edge plate joint welded at the same rpm. The tensile strength of coated edge plate joint welded at 2240 rpm was found that 4% higher than the plate welded at 1400 rpm. Moreover, the non-coated edge plate welded at 1400 rpm showed 11.65% higher than the plate welded at 2240 rpm.

**Table 2:** Tensile and flexure strength.

S.No	FSW joint	Sample	Tensile Strength (MPa)	Average tensile strength (MPa)	% Elongation	Average% elongation	Flexure strength (MPa)	Average flexure strength (MPa)
1	Coated edge plate (rpm 1400)	1	272.83	272.69	29.84	30.55	35.56	34.53
		2	272.80		31.50		33.61	
		3	272.46		30.30		34.44	
2	Coated edge plate (rpm 2240)	1	280.33	283.74	31.43	31.42	25.69	25.64
		2	287.50		31.40		26.53	
		3	283.40		31.42		24.72	
3	Non coated edge plate (rpm 1400)	1	187.66	190.92	11.43	12.46	11.97	12.49
		2	195.00		13.56		11.46	
		3	190.10		12.4		14.028	
4	Non coated edge plate (rpm 2240	1	171.16	170.99	10.53	11.35	11.87	12.71
		2	170.83		12.28		14.31	
		3	171.00		11.23		11.94

The results of percentage elongation of welded joints show that incorporation of TiB2 in the friction weld zone decreased the percentage elongation of joints due to brittle behavior developed in the friction stir zone which was observed during the testing and visually inspected of tested samples as shown in Fig. 11. Figure 11 indicates that the length of tested samples of the non-coated edge is longer than the coated edge joint welded at all rpm.

Table 2 and Fig. 12 revealed that the percentage elongation of coated edge plate welded joint is lower than the non-coated edge plate joints. The percentage elongation of coated edge joint was observed approximately 1.5 times lower than the non-coated edge plate joint.

**Figure 13:**
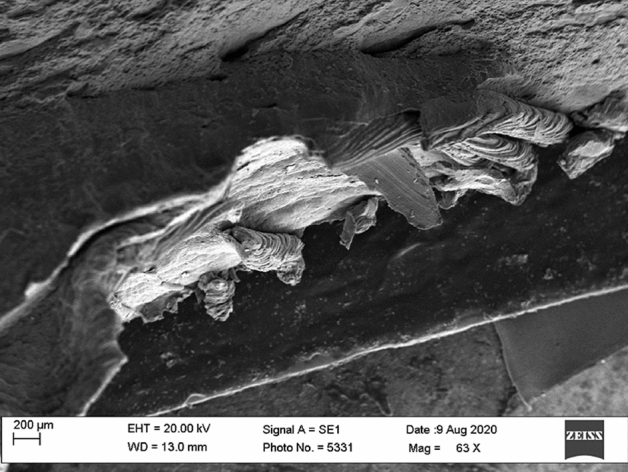
SEM image of non-coated welded joints.

**Figure 14:**
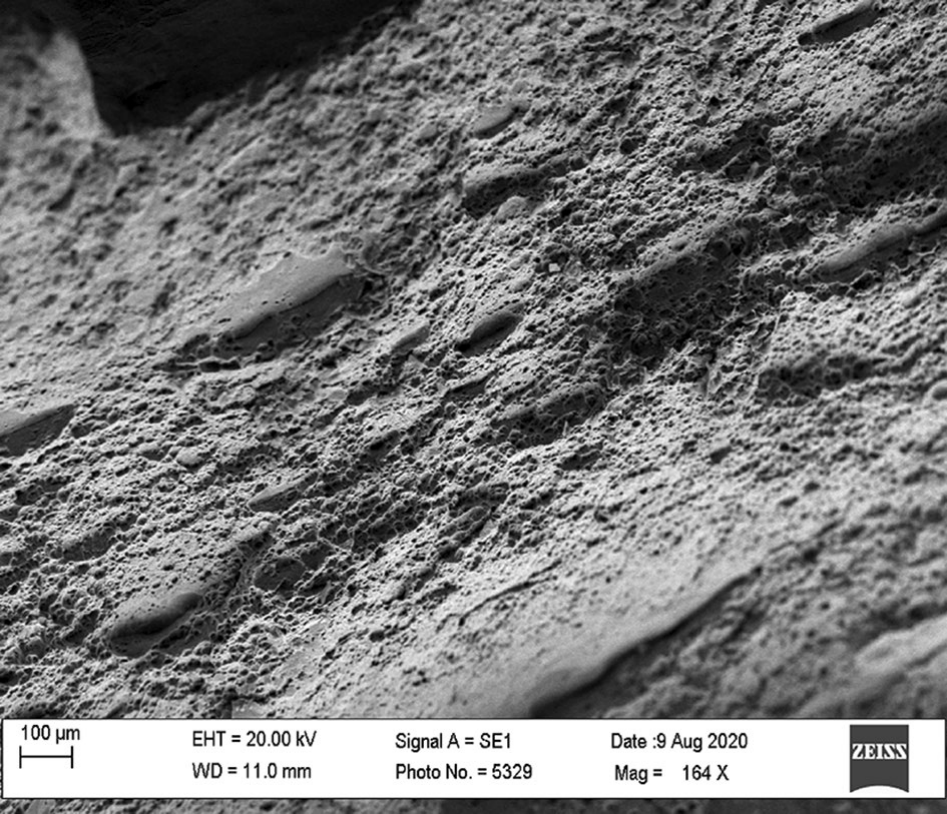
SEM image of TiB_2_ coated welded joint.

The fractography of tensile tested samples of Non-coated and TiB_2_ coated welded joints was carried out using scanning electron microscopy (SEM). The SEM images of testing samples are shown in Figs. 13 and 14. Figure 13 reveals an elongated fibrous fracture which seems like a ductile fracture whereas Fig. 14 which is TiB2 coated joint showed a shortened fibrous fracture and intergranular cleavage which resembles the brittle nature of the fracture.

**Figure 15:**
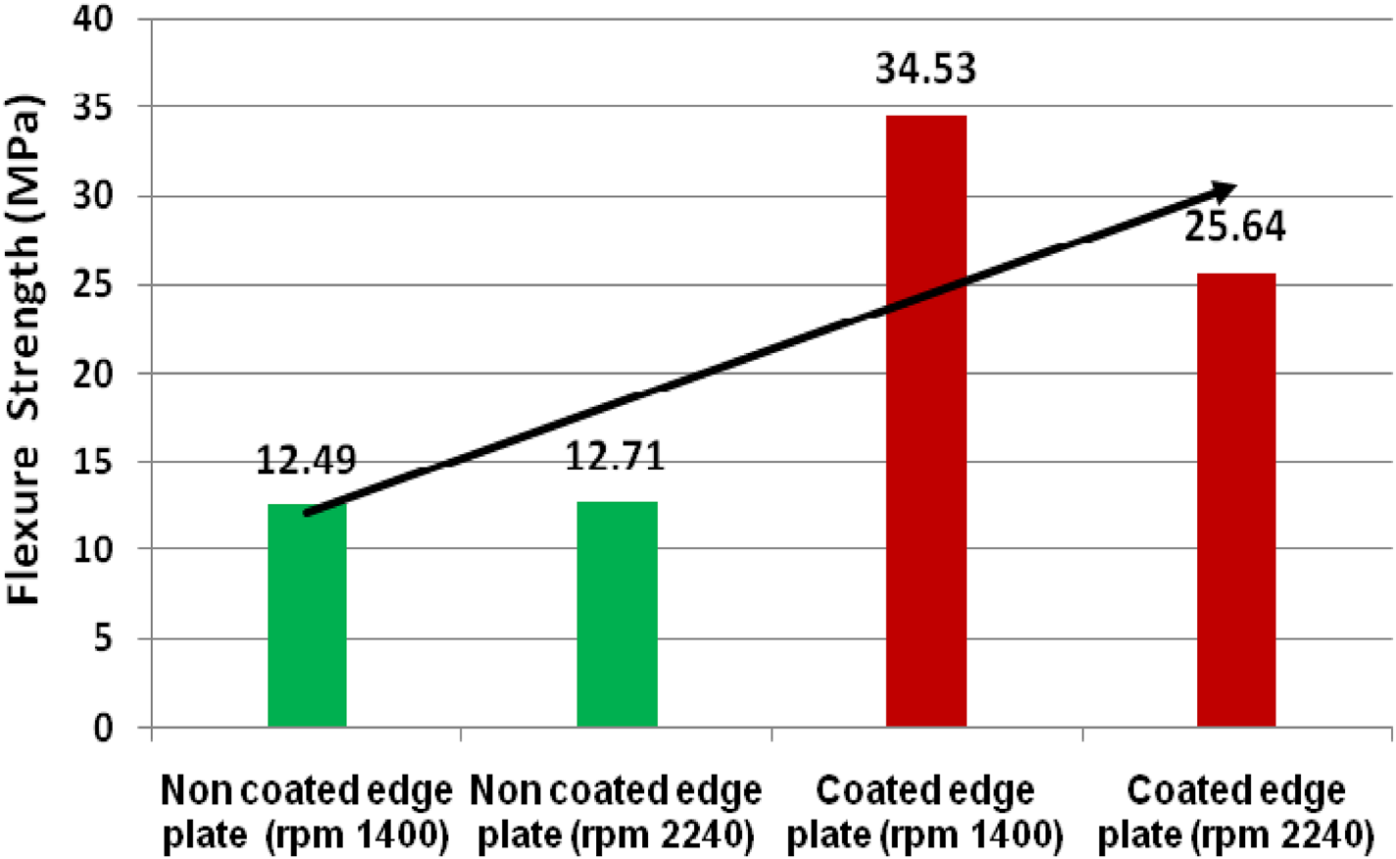
Flexure strength of welded joints at varying rpm.

The flexure strength of welded joints is depicted in Fig. 15. Figure 15 shows that the flexure strength of TiB2 reinforced joints fabricated at all rpm is higher compared to the unreinforced joints^32,33^. Furthermore, it was found that the increase in rpm does not scale up the flexure strength of unreinforced joints. However, reinforcing TiB2 in the weld zone enhanced the flexure strength of joints. Moreover, the flexure strength of TiB2 reinforced joints fabricated at 1400 rpm was found higher than the fabricated at 2240 rpm which was found 34.67% superior to the unreinforced joints fabricated at the same rpm. The flexure strength of TiB2 reinforced joints fabricated at 2240 rpm was found to be approximately double in comparison to non-coated edge plate joints fabricated at the same rpm. Moreover, the flexure strength of TiB2 reinforced joints fabricated at 1400 rpm was found approximately 1.5 times higher as compared to non-coated edge plate joints fabricated at the same rpm.

### Impact strength and hardness

**Figure 16:**
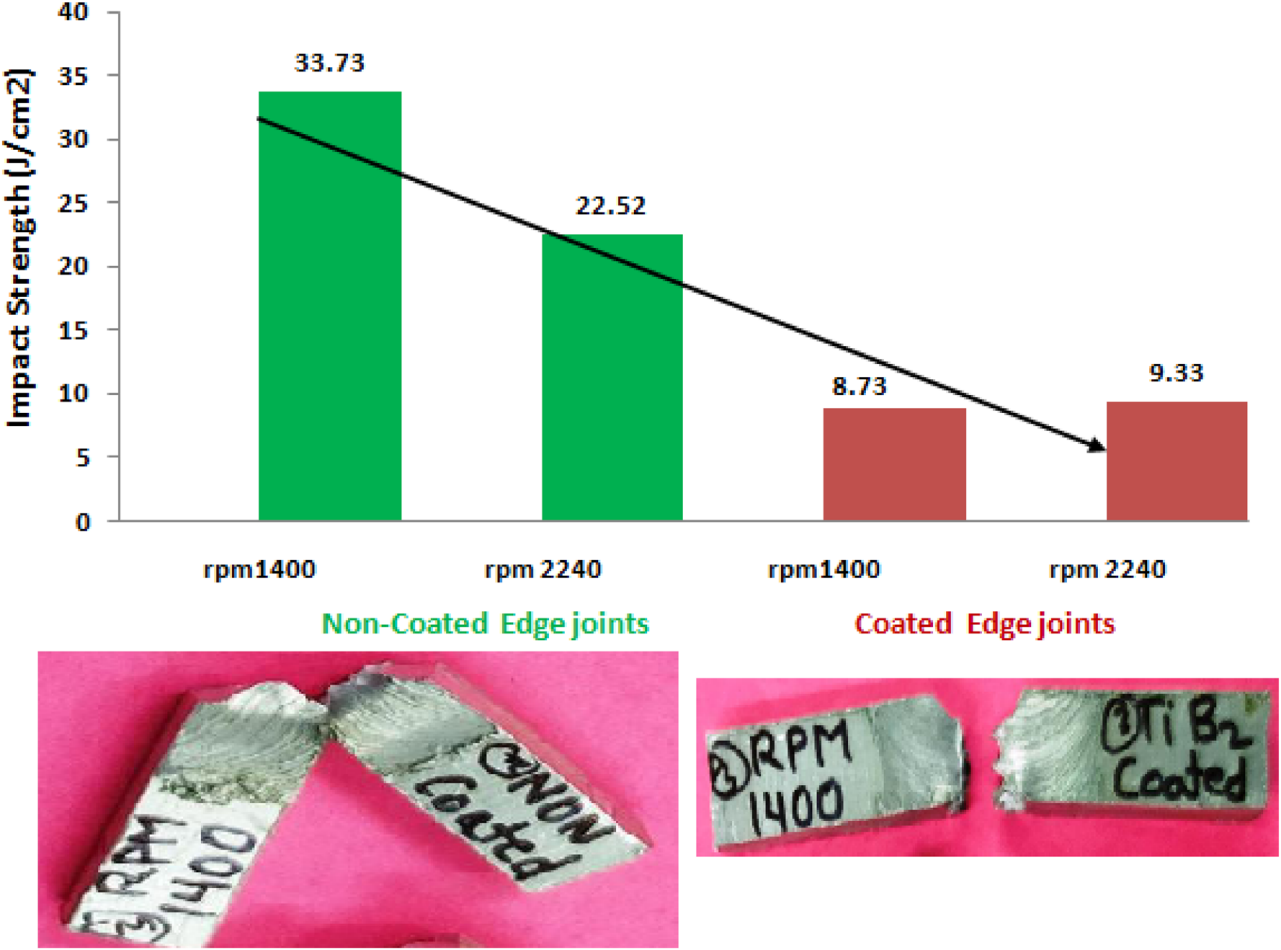
Impact strength of welded joints at varying rpm.

The Charpy impact testing data of all category samples are reported in Table 3 and depicted in Fig. 16. The three samples of each category were tested. Figure 16 showed that reinforcement of TiB2 in the friction stir weld zone decreased the impact strength of the joint. Furthermore, the impact strength of non coated edge plate joint welded at 1400 rpm was found higher than the welded at 2240 rpm which was 49.77% superior. Moreover, the impact strength of TiB2 coated edge joints was approximately the same welded at all rpm. Furthermore, the impact strength of TiB2 reinforced joints was found around three times lower than the unreinforced welded joints. The lower impact strength of reinforced joints might be due to the brittle properties of joints because of TiB2 reinforcement in the weld zone^37^.

**Table 3:** Impact strength of joints.

S No	rpm	Sample no	Impact strength (Joule/cm^2^)	Mean impact strength (Joule/cm^2^)
**Non-coated edge plate joints**				
1	1400	1	35.2	33.73
		2	32.9	
		3	33.1	
2	2240	1	23.7	22.52
		2	21.5	
		3	22.4	
**Coated edge plate joints**				
3	1400	1	9.2	8.73
		2	8.6	
		3	8.4	
4	2240	1	9.2	9.33
		2	8.9	
		3	9.9

**Figure 17:**
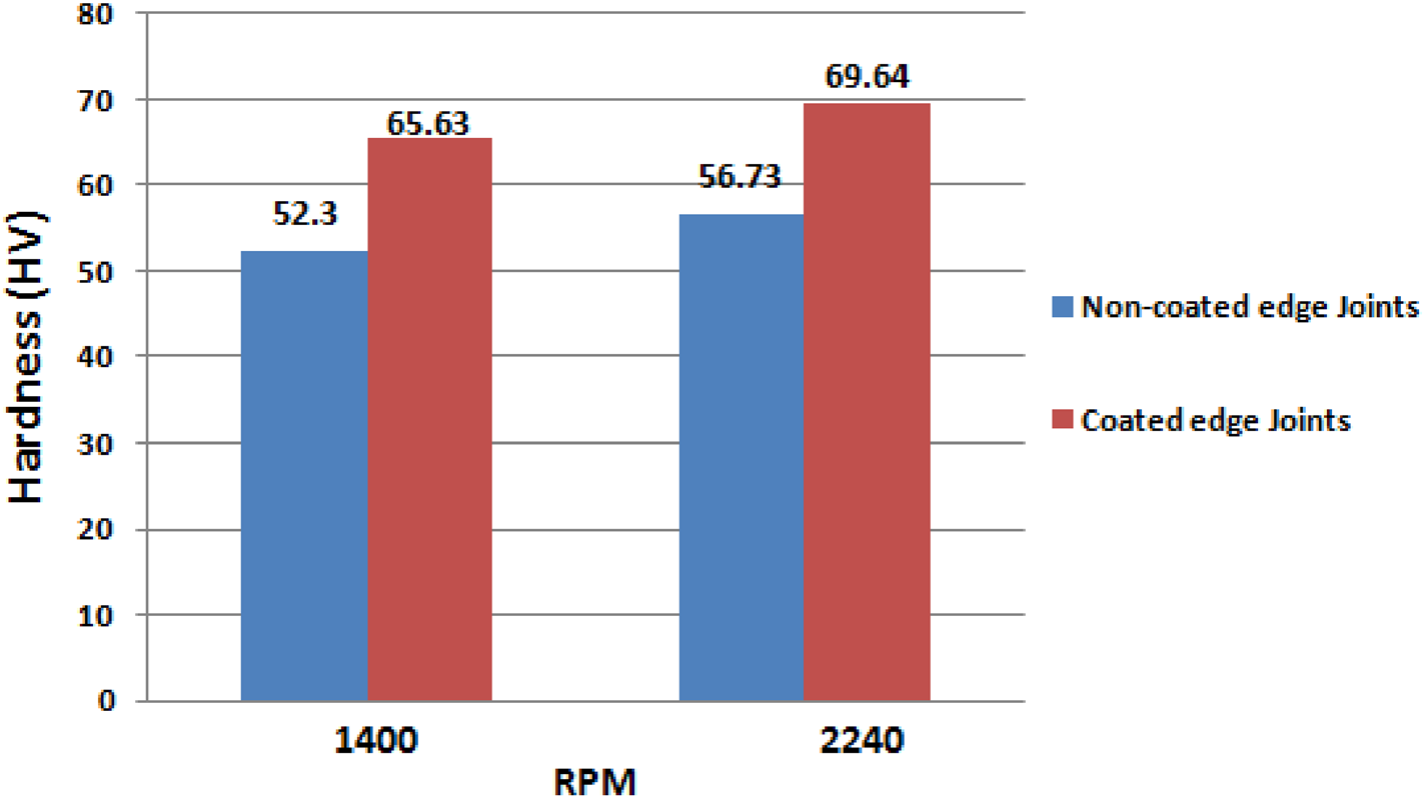
Hardness of welded joint.

During testing of coated edge plate joints, it was observed that samples break completely into two pieces. However, non-coated edge joints and sample breaking edges adhere to each other as shown 16. The brittle and ductile fracture behavior of the impact-tested sample is shown in Fig. 16. The hardness test results are reported in Table 4 and depicted in Fig. 17. Figure 17 showed that the hardness of coated edge joints is higher than that of non-coated edge joints. Furthermore, reinforcement of TiB2 in the friction stir zone and increase of rpm increased the hardness of welded joints^29,32,33^. Moreover, TiB2 coated edge joints revealed 22.75% higher hardness than the non-coated joints welded at the rpm 2240. However, non-coated edge joints exhibited 8.47% higher harness as compared to welded at rpm of 1400. However, coated edge plate joints at the rpm of 2240 showed 6.11% superior hardness than the welded at the rpm of 1400.

**Table 4:** Hardness of joints.

S.No	FSW joint	RPM	Sample	Hardness(HV)	Average hardness (HV)
1	Non-coated edge Joints	1400	1	53.4	52.30
			2	48.3	
			3	55.0.2	
		2240	1	55.7	56.73
			2	58.1	
			3	56.4	
2	Coated edge Joints	1400	1	67.4	65.63
			2	64.3	
			3	65.0.2	
		2240	1	70.3	69.64
			2	68.7	
			3	69.9

## Conclusion

1. TiB2 reinforced and without reinforced joints were successfully fabricated using the friction stir welding process.
2. The tensile strength and flexure strength of TiB_2_ reinforced joints were found higher than the unreinforced joints. However, the percentage elongation of TiB_2_ reinforced joints was found lower than the unreinforced joints.
3. The tensile strength of TiB_2_ reinforced joints welded at 2240 rpm was found 283.74 MPa which is 39.74% superior that the non-coated edge plate.
4. The flexure strength of TiB_2_ reinforced joints was found 34.53 MPa and 25.64 MPa fabricated at 2240 and 1400 rpm respectively.
5. The percentage elongation of coated edge and non-coated edge joint were observed 12.46 and 11.35, and 30.55 and 31.42 fabricated at 1400 and 2240 rpm respectively which is approximately 1.5 times lower than the non-coated edge plate joint.
6. Impact strength of TiB_2_ coated and non-coated edge joints were found 33.73, 22.52, 8.73, and 9.33 J/cm^2^ respectively. The impact strength of TiB_2_ coated edge joints decreased.
